# Evaluation of a Human BCG Challenge Model to Assess Antimycobacterial Immunity Induced by BCG and a Candidate Tuberculosis Vaccine, MVA85A, Alone and in Combination

**DOI:** 10.1093/infdis/jit647

**Published:** 2013-11-23

**Authors:** Stephanie A. Harris, Joel Meyer, Iman Satti, Leanne Marsay, Ian D. Poulton, Rachel Tanner, Angela M. Minassian, Helen A. Fletcher, Helen McShane

**Affiliations:** Jenner Institute, University of Oxford, Oxford, United Kingdom

**Keywords:** tuberculosis, human BCG challenge, vaccine, MVA85A

## Abstract

***Background.*** A new vaccine is urgently needed to combat tuberculosis. However, without a correlate of protection, selection of the vaccines to take forward into large-scale efficacy trials is difficult. Use of bacille Calmette-Guérin (BCG) as a surrogate for human *Mycobacterium tuberculosis* challenge is a novel model that could aid selection.

***Methods.*** Healthy adults were assigned to groups A and B (BCG-naive) or groups C and D (BCG-vaccinated). Groups B and D received candidate tuberculosis vaccine MVA85A. Participants were challenged with intradermal BCG 4 weeks after those who received MVA85A. Skin biopsies of the challenge site were taken 2 weeks post challenge and BCG load quantified by culture and quantitative polymerase chain reaction (qPCR).

***Results.*** Volunteers with a history of BCG showed some degree of protective immunity to challenge, having lower BCG loads compared with volunteers without prior BCG, regardless of MVA85A status. There was a significant inverse correlation between antimycobacterial immunity at peak response after MVA85A and BCG load detected by qPCR.

***Conclusion.*** Our results support previous findings that this BCG challenge model is able to detect differences in antimycobacterial immunity induced by vaccination and could aid in the selection of candidate tuberculosis vaccines for field efficacy testing.

***Clinical Trials Registration*** NCT01194180.

Disease caused by *Mycobacterium tuberculosis* continues to be a major global health problem. In 2011, 8.7 million new cases of tuberculosis were diagnosed worldwide and 1.4 million people died from the disease [1]. With tuberculosis causing a quarter of the deaths in people living with human immunodeficiency virus (HIV) [1] and with the emergence of increasingly drug-resistant strains of *M. tuberculosis*, an effective vaccine is urgently needed now in order to reduce the burden of this disease.

Since 2002, more than a dozen candidate vaccines have been entered into clinical testing [2]. However, it is difficult to determine which of these candidates will progress from relatively small-scale safety and immunogenicity studies through to large-scale, expensive efficacy trials because an immune correlate of vaccine-induced protection against infection or disease does not exist. Preclinical animal challenge models of *M. tuberculosis* infection [3–6] and in vitro mycobacterial killing assays [7–9] are used to assess vaccine efficacy. However, it is not clear whether either of these reliably predict what occurs in vivo in humans. Thus, the evaluation of vaccine efficacy currently relies on large, expensive, and time-consuming efficacy trials. A human mycobacterial challenge model that could be used to assess the efficacy of candidate tuberculosis vaccines at an early stage would be a great advancement to the field. Human challenge models are routinely used in vaccine development for pathogens such as malaria, influenza, dengue fever, and typhoid [10–13]; however, the deliberate infection of humans with *M. tuberculosis* would not be ethically acceptable. Previously, we demonstrated that a novel human challenge model that uses bacille Calmette-Guérin (BCG) as a surrogate for *M. tuberculosis* infection can detect differences in antimycobacterial immunity induced by previous BCG vaccination [14]. In this earlier trial, healthy volunteers were challenged with intradermal BCG. The BCG load was then quantified from a skin biopsy at the challenge site at 1, 2, and 4 weeks post challenge by culture on solid agar and quantitative polymerase chain reaction (qPCR). It was found that optimum recovery of BCG was achieved in 2 weeks; this time period was chosen for future challenge studies. Here we use this BCG challenge model to evaluate the reduction in mycobacterial load induced by BCG alone; a candidate tuberculosis vaccine, MVA85A; and a BCG prime–MVA85A boost vaccine regimen.

## METHODS

### Trial Design

**Figure 1.**
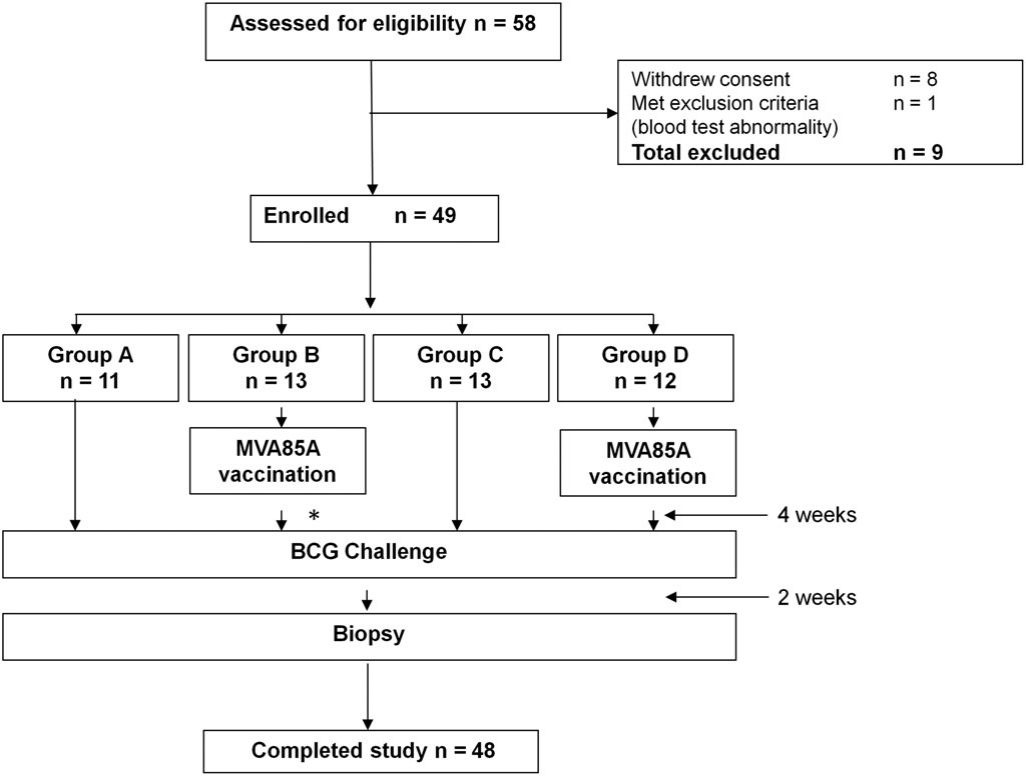
Consort diagram showing participant recruitment and follow-up. *One volunteer withdrew from group B for personal reasons after MVA85A vaccination but before bacille Calmette-Guérin challenge.

This phase 1 trial (ClinicalTrial.gov registry NCT01194180) was approved by the Medicines and Healthcare Products Regulatory Agency (EudraCT 2010-018425-19) and the Oxfordshire Research Ethics Committee A (reference 10/H0505/31). Twenty-six BCG-vaccinated and 23 BCG-naive healthy volunteers aged 18–55 years were enrolled between March 2011 and November 2011 at the Centre for Clinical Vaccinology & Tropical Medicine, Churchill Hospital, Oxford, United Kingdom (see Figure 1). All participants gave written informed consent, and the trial was conducted according to the principles of the Declaration of Helsinki and Good Clinical Practice.

Those enrolled were in good health, had normal baseline hematology and biochemistry, and were serologically negative for hepatitis B, hepatitis C, and HIV. Latent infection with *M. tuberculosis* was excluded by a negative ex vivo enzyme-linked immunosorbent spot (ELISpot) assay response to ESAT-6 and CFP-10 peptides. The reasons for exclusion of 9 participants are shown in Figure 1. One individual in group B had to withdraw from follow-up for personal reasons after receiving MVA85A but before BCG challenge and was therefore replaced.

### Treatment Groups

Participants were assigned to group A (BCG-naive; no vaccine received), group B (BCG-naive at baseline; received intradermal MVA85A, dose 1 × 10^8^ pfu), group C (BCG-vaccinated at baseline; median time since vaccination 10 years), or group D (BCG-vaccinated at baseline; median time since vaccination 10.5 years; received intradermal MVA85A, dose 1 × 10^8^ pfu) based on their prior BCG vaccination status and meeting inclusion criteria. One volunteer who was enrolled into group A on the basis of negative BCG status was later reassigned to group C after discovering that he/she had, in fact, received BCG as an infant (Figure 1).

### Vaccine

Clinical-grade MVA85A was constructed as previously described [15] and produced following good manufacturing practices by IDT Biologika GmbH (Dessau-Rosslau, Germany).

### Challenge

All participants were challenged with a standard vaccine dose of intradermal BCG (SSI (Statens Serum Institut); 0.1 mL containing 2 to 8 × 10^5^ CFU). Those in groups B and D were challenged 4 weeks after MVA85A vaccination. To minimize variation between BCG vials, as many volunteers as possible were challenged from the same vial of BCG within 2 hours of reconstitution (15 different BCG vaccine vials were used over the course of the trial to challenge 48 volunteers). The challenge dose was verified by plating serial dilutions of a 100-µL aliquot onto solid Middlebrook 7H10 agar (Sigma).

### Skin Biopsies

Skin biopsies were performed on the BCG challenge site of all 48 volunteers by a single operator 2 weeks post challenge as previously described [14]. The 4-mm punch biopsy specimen was taken from the center of the BCG vaccination site, transferred to a sterile Cryovial, snap frozen on dry ice, and stored in liquid nitrogen until the day of processing.

### Biopsy Homogenization and Culture

All 48 biopsies were processed on the same day. Samples were thawed in a 37°C water bath and transferred to a Dispomix tube (Miltenyl Biotech) that contained 1 mL sterile phosphate-buffered saline (PBS). Tubes were loaded onto a Dispomix machine (Thistle Scientific) and homogenized as previously described [16]. Next, 100 µL of neat homogenate and 100 µL of a 10^−1^ and 10^−2^ dilution were plated in triplicate onto Middlebrook 7H10 agar and incubated at 37°C for 5 weeks. A BCG SSI vaccine vial was reconstituted in PBS and 100 µL of a 10^−2^, 10^−3^, and 10^−4^ dilution were plated in triplicate as positive controls. The remaining biopsy homogenate was stored at −20°C for later DNA extraction.

### DNA Extraction

Homogenate was thawed and BCG DNA from 200 µL homogenate was released using the tough microorganism lysing kit (Precellys) in a Precellys 24 machine at 6500 rpm for 3 × 30 seconds. Homogenate was transferred to a separate tube, and 50 µL PBS was used to wash the remaining homogenate from the beads. Next, 180 µL animal tissue lysis buffer and 20 µL proteinase K (Qiagen) were added, vortexed, and incubated at 56°C for 4 hours. From this point, the extractions were carried out as previously described [16].

### qPCR

Primers ET 1 and ET 3 were used for detection of BCG DNA. These are complementary to regions that flank the BCG deletion RD1 sequence and amplify a 196-bp fragment [17]. These sequences were modified by Minassian et al [16], and the modified sequences were used for this work (Table 1). PCR reactions were carried out as previously described [16] using BCG-naive macaque tissue homogenate as a negative control. A standard curve was obtained by extracting BCG DNA from 1 in 10 serial dilutions of 5 pooled vaccine vials in PBS and correcting for live BCG from the corresponding colony-forming unit counts on solid agar.

**Table 1.** Primer Sequences Used to Detect Bacille Calmette-Guérin by Quantitative Polymerase Chain Reaction

Primer	Primer Sequence
ET 1/3 forward	5′ -CCG CCG ACC GAC CTG ACG AC- 3′
ET 1/3 reverse	5′ -GGC GAT CTG GCG GTT TGG GG- 3′

### Ex vivo Interferon-gamma ELISpot Assay

Peripheral blood mononuclear cells (PBMCs) were isolated from whole blood and ELISpots were performed as previously described [18]. This occurred for all volunteers on the day of screening, the day of BCG challenge, and the day of skin biopsy. Groups B and D also had an ELISpot performed 7 days after MVA85A vaccination, which occurred 21 days prechallenge. Responses to purified protein derivative (PPD) from *M. tuberculosis* (SSI; 20 µg/mL) and a single pool of 66 Ag85A peptides (Peptide Protein Research; 2 µg/mL each peptide) were assessed for all volunteers at each time point. Staphylococcal enterotoxin B (Sigma) was used as a positive control (10 µg/mL). Unstimulated PBMCs were used as a measure of background interferon-gamma (IFN-γ) production. Results are reported as spot-forming cells (SFC) per million PBMC, calculated by subtracting the mean count of the unstimulated PBMCs from the mean count of duplicate antigen wells and correcting for the number of PBMCs in the well.

### Whole Blood Growth Inhibition Assay

The whole blood growth inhibition assay was performed on heparinized whole blood on the day of BCG challenge using the BACTEC mycobacteria growth indicator tube (MGIT) system (Becton Dickinson) as previously described [19], with the exception that whole blood was incubated with BCG (Pasteur) for 96 hours instead of 72 hours. Growth inhibition was determined by calculating time to positivity (TTP) in the sample and TTP in the control and converting to colony-forming units using a standard curve. The growth ratio (GR) was calculated by GR = CFU sample (96 hours)/CFU control (0 hours).

### Swabbing of BCG Vaccination Site

The feasibility of swab-based quantification of BCG from the surface of the BCG challenge vaccination site, as an alternative or complementary technique to the biopsy technique, was investigated in a separate cohort of healthy volunteers (study approved by the University of Oxford Central University Research Ethics Committee, reference MSD/IDREC/C1/2012/7). Seven BCG-naive, adult healthcare workers who were due to receive BCG vaccination for employment reasons were recruited and gave written informed consent. Cotton-tipped swabs (Transwab MW171, Medical Wire & Equipment) were used to swab the surface of the BCG site at 2, 7, 14, and 21 days following vaccination. The ability of this method to recover BCG from the swabs was tested before the study began. Serial dilutions of a BCG vaccine vial (SSI) were made and 100 µL of each dilution was plated onto Middlebrook 7H10 agar. Twenty microliters of each dilution was used to spike the swabs, which were then immersed in 500 µL Middlebrook 7H9 broth (Sigma), left for 1 hour, and sonicated for 30 seconds. 300 µL of broth was then plated onto Middlebrook 7H10 agar and incubated at 37°C for 3 weeks. The number of colonies recovered was compared with the inoculum in 20 µL. Swabs from the BCG vaccination site were processed in the same way.

### Statistical Analysis

Statistical analyses were performed using GraphPad Prism. One-way analysis of variance (Kruskal-Wallis) and Mann–Whitney *U* tests were used to determine significant differences between groups. The Wilcoxon matched pairs test was used to determine differences between time points in the same group. The Spearman rank correlation test was used to determine correlations between numbers of BCG recovered from biopsies and ex vivo IFN-γ ELISpot responses.

## RESULTS

### BCG Challenge Was Safe and Well Tolerated by All Groups

Other than BCG vaccination status, the volunteers’ baseline characteristics did not significantly differ among the 4 groups (Table 2). BCG challenge was well tolerated, with all volunteers developing an expected local inflammatory reaction to BCG. It was noted that previously BCG-vaccinated volunteers (groups C and D) experienced significantly more frequent local adverse events and significantly greater diameters of erythema and swelling at the challenge vaccination site during the first 2 weeks than did BCG-naive volunteers (groups A and B; data not shown), which is consistent with previous studies [20, 21]. Intradermal administration of candidate vaccine MVA85A at a dose of 1 × 10^8^ pfu was safe and well tolerated, with an adverse event profile consistent with previous experience [18, 22]. No serious adverse events occurred. Vaccination with MVA85A 4 weeks before challenge had no effect on the reactogenicity of the subsequent BCG challenge.

**Table 2.** Demographics of Enrolled Participants

Characteristic	Group A (n = 11)	Group B (n = 12)	Group C (n = 13)	Group D (n = 12)	*P* Value
Prior BCG	No	No	Yes	Yes	
MVA85A	No	Yes	No	Yes	
Female, n (%)	8 (73)	7 (58)	6 (46)	6 (50)	0.58
Median age, years (range)	23 (18–41)	23 (19–30)	23 (21–41)	22 (19–33)	0.63
Median time interval since BCG in years (range)	n/a	n/a	10 (8–38)	10.5 (6–33)	0.48
Continent of birth					
Europe	9	8	13	10	
Africa	0	0	0	1	
Asia	0	0	0	1	
Americas	1	3	0	0	
Australasia	1	1	0	0	

Abbreviations: BCG, bacille Calmette-Guérin; n/a, not applicable.

### BCG Was Detected by Both qPCR and Culture

**Figure 2.**
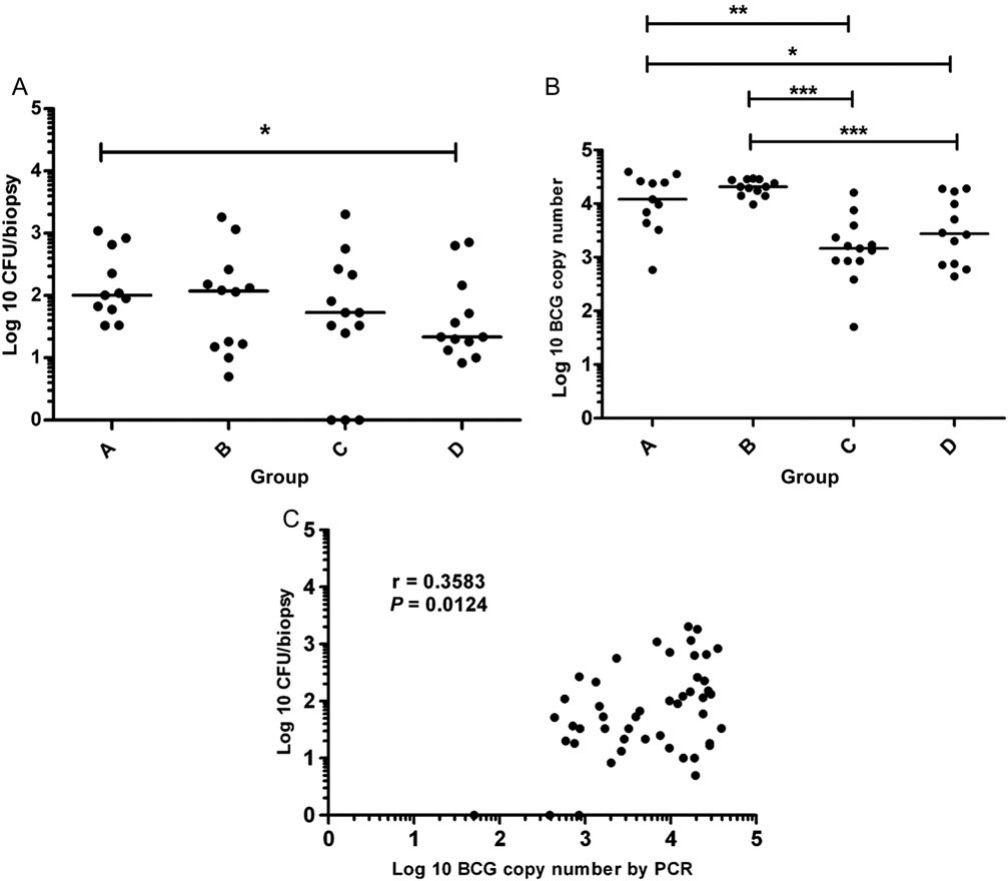
Quantification of bacterial load from punch biopsies 14 days post bacille Calmette-Guérin (BCG) challenge by culture on solid agar (*A*) and quantitative polymerase chain reaction (qPCR) (*B*). Individual values are shown for each volunteer. Horizontal bars indicate median values in each group. Significant differences between groups are as follows: *P ≤ .05, **P ≤ .01, ***P ≤ .001; Mann–Whitney *U* test. A significant positive correlation was observed between the culture and qPCR results (*C*).

BCG was detected in all 48 biopsy samples by qPCR and in 45 of 48 samples by culture on solid agar (Figure 2). Estimated copy numbers per biopsy using PCR were 1–2 logs higher than the corresponding colony-forming unit counts by culture. A positive correlation was observed between the 2 methods of detection (Spearman R = 0.36, *P* = .01; Figure 2).

### BCG Challenge Dose Administered Was Comparable for All Volunteers

Quantification of BCG from each vaccine vial used in this trial showed that the range in challenge dose was small (1.85 × 10^5^ to 3.15 × 10^5^ cfu, median = 2.35 × 10^5^ cfu) and at the lower end of that stated by the manufacturer [23].

### Levels of BCG Recovered Were Lower in Groups With Previous BCG Vaccination

Enumeration of BCG by solid culture showed a trend toward a lower median colony-forming unit count in the previously BCG-vaccinated groups, with a statistically significant 0.5-log reduction in colony-forming unit count between group A (naive) and group D (BCG–MVA85A; *P* = .02, Mann–Whitney *U* test; Figure 2). Using PCR, there was a significant 0.5- to 1-log reduction in estimated BCG copy number between the BCG-naive (A or B) and BCG-vaccinated groups (C or D; Mann–Whitney *U* test). No further reduction in BCG numbers was detected after vaccination with MVA85A.

### Ex Vivo IFN-γ ELISpot Responses Were as Expected for Vaccination Schedule Received

**Figure 3.**
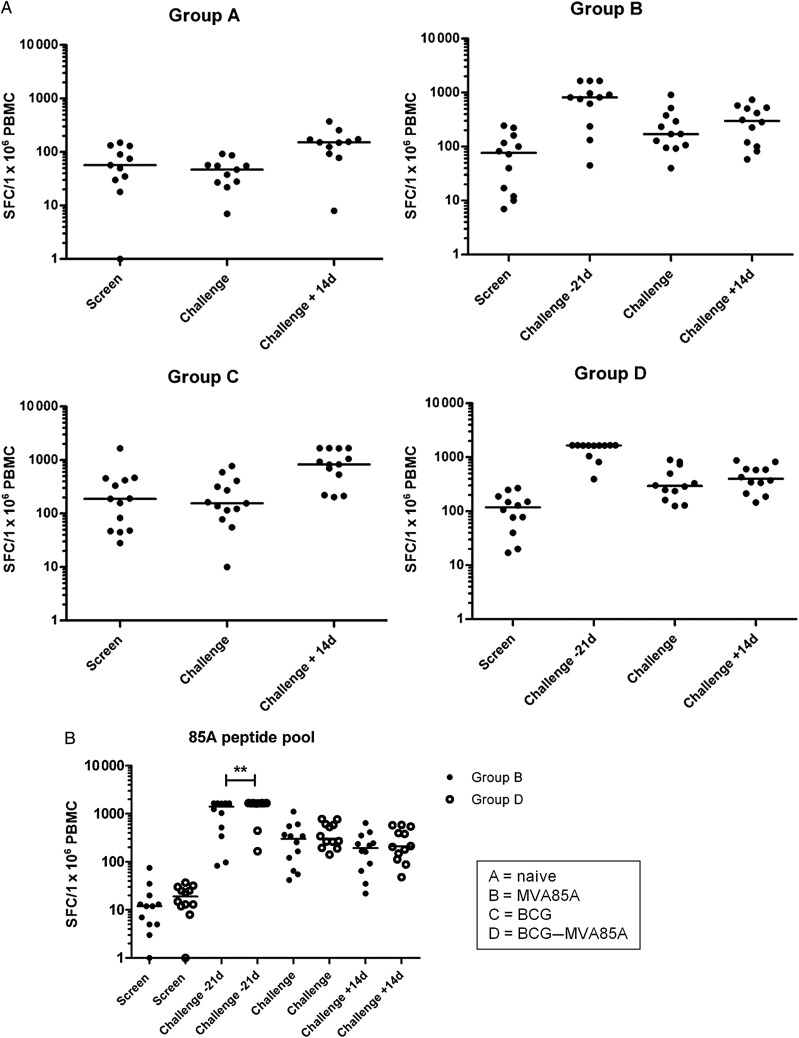
Ex vivo interferon-gamma enzyme-linked immunosorbent spot (ELISpot) assay responses to purified protein derivative from *Mycobacterium* *tuberculosis* for all groups (*A*) and to a single pool of Ag85A peptides for groups B and D (*B*). A value of 1667 spot-forming cells (SFCs)/1 × 10^6^ peripheral blood mononuclear cells (PBMCs) represents a blackout in the ELISpot well. ** *P* < .01. Abbreviation: BCG, bacille Calmette-Guérin.

Ex vivo IFN-γ ELISpot responses to PPD and a single pool of Ag85A peptides are shown in Figure 3. There were no significant differences in baseline Ag85A responses among the 4 groups (data not shown for groups A and C); however, baseline PPD responses were significantly higher (*P* = .008, Mann–Whitney *U* test) in the previously BCG-vaccinated groups. Those in group D who received MVA85A as a boost to BCG had significantly higher responses to Ag85A 7 days post vaccination than those in group B who were BCG naive, as previously reported [24]. However, Ag85A responses between the 2 groups were not significantly different on the day of challenge or on the day of biopsy. Responses to PPD on the day of challenge were significantly lower in group A compared with each of the other 3 groups; however, there was no significant difference in responses among groups B, C, and D at this time point. PPD responses for those in groups A and C were significantly higher 14 days post challenge than for those measured on the day of challenge (*P* = .002 and .0002, respectively; Wilcoxon matched-pairs). This significant increase was not observed in groups B and D due to the confounding effect of recent vaccination with MVA85A.

### Ex Vivo IFN-γ ELISpot Responses Correlate With Number of BCG Detected

**Figure 4.**
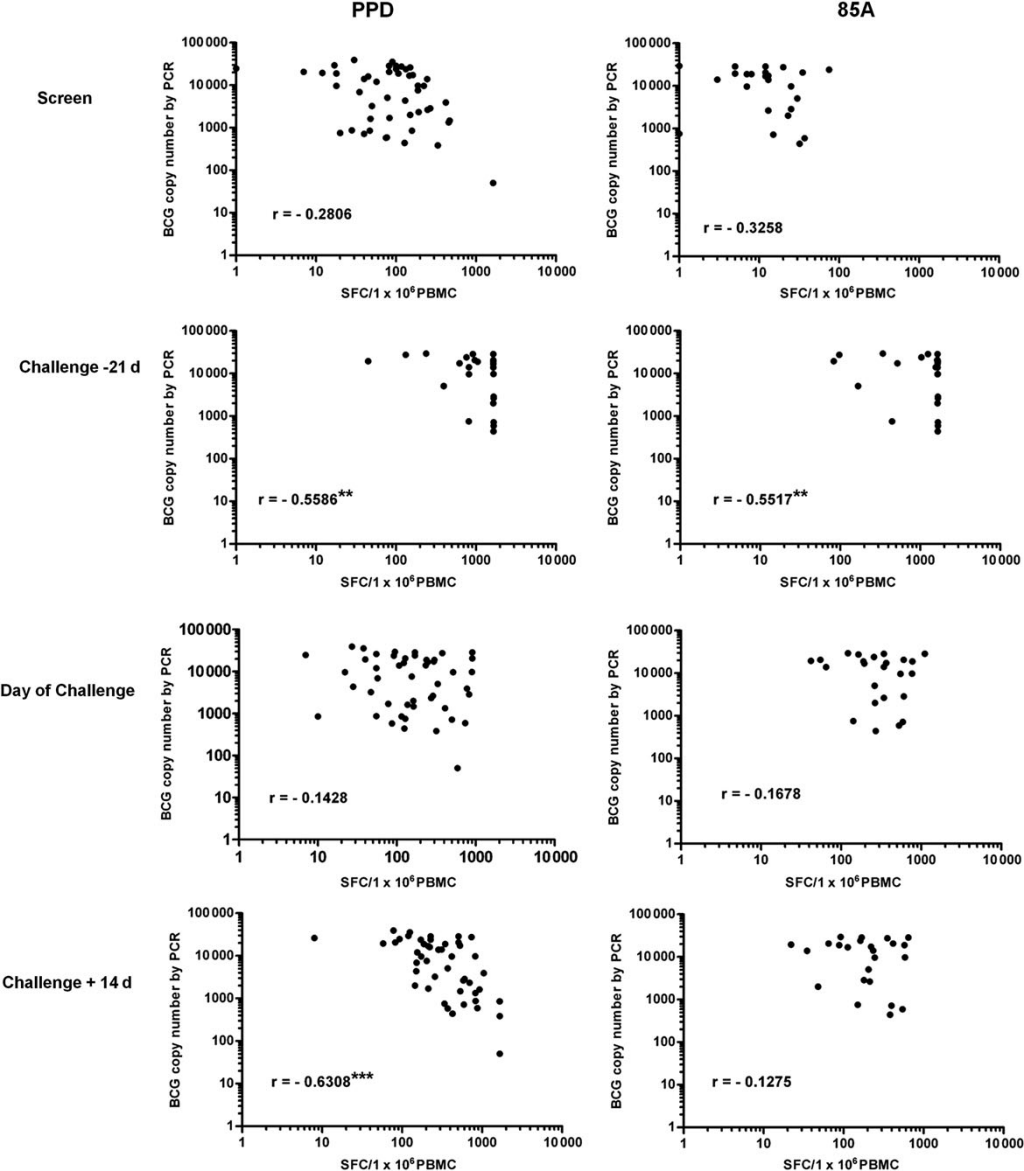
Correlation between ex vivo interferon-gamma enzyme-linked immunosorbent spot assay responses to purified protein derivative or Ag85A and estimated bacille Calmette-Guérin (BCG) copy number by polymerase chain reaction (PCR). Spearman R values are shown with asterisks indicating *P* values as follows: *P ≤ .05, **P ≤ .01, ***P ≤ .001. Abbreviations: PBMC, peripheral blood mononuclear cell; SFC, spot-forming cell.

The correlation between ex vivo IFN-γ ELISpot responses and BCG colony number detected by PCR is shown in Figure 4. An inverse correlation was observed at all time points for responses to PPD and Ag85A, and this correlation is significant for both antigens 7 days after receipt of MVA85A (*P* = .005, Spearman) and for PPD 14 days post challenge (*P* = <.0001, Spearman). The same trend was observed when ELISpot responses were correlated with colony-forming unit counts by culture but did not reach statistical significance at any time point (data not shown).

### In Vitro Mycobacterial Growth Inhibition Did Not Differ Among Groups on Day of Challenge

**Figure 5.**
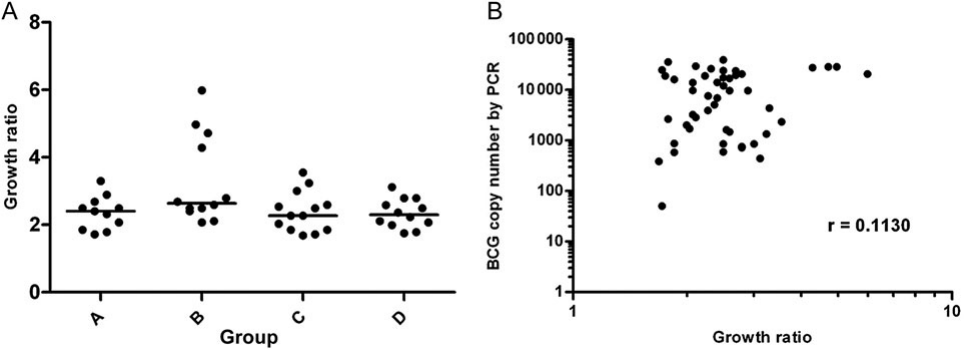
Growth ratios obtained from the mycobacteria growth indicator tube assay (*A*) and correlation with estimated bacille Calmette-Guérin (BCG) copy number by quantitative polymerase chain reaction (PCR) (*B*).

Figure 5 shows the growth ratios obtained from incubating whole blood taken on the day of challenge with BCG in the MGIT assay. This assay detected no significant differences among the 4 treatment groups in the ability of whole blood to reduce growth of BCG during a 96-hour incubation period (*P* = .13, Kruskal-Wallis). A nonsignificant positive correlation was observed between growth ratio and BCG copy number by PCR (Figure 5*B*) and also between growth ratio and colony-forming unit count (data not shown).

### BCG Was Not Detected From Swabbing of the BCG Vaccination Site

BCG could not be detected by swabbing of the vaccination site in any of the volunteers at any of the time points investigated, by either culture on solid agar or qPCR. This is despite an average recovery of 88% by culture when swabs were spiked with serial dilutions of a BCG vaccine vial.

## DISCUSSION

Here we present a proof-of-concept clinical trial to evaluate a novel BCG challenge model in BCG- and/or MVA85A-vaccinated adults.

In this trial, 48 volunteers were challenged with a standard vaccine dose of BCG. A punch biopsy of the vaccination site was taken 14 days later. BCG was detected in all 48 biopsies by qPCR and in 45 of 48 biopsies by culture on solid agar. It has been shown that estimated copy numbers using PCR were 1–2 logs higher than the corresponding colony-forming unit counts [14], even though a positive correlation was observed between the 2 methods of detection. The discrepancy between the 2 methods of quantification is most likely due to the fact that PCR does not distinguish between live and dead BCG, whereas culture only detects viable bacteria. The challenge dose received by each volunteer was similar and no correlation was observed between challenge dose and BCG recovery. BCG could not be detected by either culture or qPCR after swabbing of the BCG vaccination site in a separate cohort.

A significant reduction in BCG was detected by solid culture in the BCG–MVA85A group compared with the naive group. Using PCR, there was a significant 0.5- to 1-log reduction in BCG copy number in the 2 groups that had been BCG vaccinated when compared with the 2 BCG-naive groups. These findings suggest that prior BCG vaccination gives some protection against a subsequent challenge dose. Administration of MVA85A 4 weeks prior to BCG challenge had no added effect on the reduction of numbers of BCG detected. This finding is consistent with data recently published on the efficacy of MVA85A in a phase 2b trial in BCG-vaccinated infants in South Africa where boosting with MVA85A conferred no significant efficacy over BCG alone [25].

Vaccination with MVA85A induced a range of ex vivo IFN-γ ELISpot responses to Ag85A 7 days post vaccination (83–1667 sfc/million PBMC, median 1649), which inversely correlated with the number of colony-forming units recovered from the punch biopsies. The same inverse correlation was observed with PPD responses from PBMC isolated 14 days post challenge, suggesting that IFN- γ produced from antigen-specific CD4+ effector T cells is important for bacterial clearance from the challenge site.

The MGIT assay did not detect any differences between the groups’ ability to reduce growth of BCG when incubated with whole blood on the day of challenge. The lack of detectable difference between the BCG-naive and BCG-vaccinated groups may be due to the length of time between BCG vaccination and when the MGIT assay was performed, that is, a median of 10 and 10.5 years for groups C and D, respectively. Other in vitro studies have shown enhanced mycobacterial growth inhibition due to BCG vaccination involving shorter time intervals (2–12 months) between vaccination and performance of the assays [26–29].

Comparison of this human BCG challenge model to animal *M. tuberculosis* challenge models shows a comparable effect of prior BCG vaccination. The same is not true of MVA85A, which has been shown to improve efficacy over BCG alone when given as a boost in preclinical animal models [5, 6, 30, 31]. However, in the animal challenge models, a high dose of *M. tuberculosis* is given by the aerosol route, while a standard vaccine dose of BCG is given by the intradermal route in the human model. Therefore, the challenge dose of BCG may be too low to detect further improvement over BCG alone. Further data are needed to truly compare the 2 models.

Data from this study support previous findings that this novel BCG challenge model can detect differences in antimycobacterial immunity induced by vaccination. In this trial, a difference could be detected between prior BCG vaccination and no prior BCG vaccination. MVA85A vaccination 4 weeks before challenge did not appear to further inhibit BCG growth. However, this study was performed in a population in which BCG had been demonstrated to be extremely effective [32]. Therefore, it might not be possible to see a additional effect of MVA85A vaccination with such small group sizes and numbers of BCG recovered. Model sensitivity needs to be improved, and further challenge trials are planned to address this issue. This will be done by varying the BCG challenge strain and the dose. The optimum time interval between vaccination and challenge also needs to be considered. After these parameters have been optimized, the study must be repeated with larger groups. The model also merits evaluation in populations where the BCG vaccine has a lower efficacy. Also, further work is needed to determine the target population and type of vaccine candidate that this BCG challenge model has utility for.

In the absence of a correlate of protection against *M. tuberculosis*, human BCG challenge provides a useful model to complement preclinical animal testing and immunological assessment to allow optimal selection of vaccines that will progress to field efficacy testing.

## References

[1] WHO Tuberculosis.

[2] Brennan MJ, Thole J (2012). Tuberculosis vaccines: a strategic blueprint for the next decade. Tuberculosis.

[3] Stylianou E, Pepponi I, Van Dolleweerd CJ, Paul MJ, Ma JK, Reljic R (2011). Exploring the vaccine potential of Dec-205 targeting in *Mycobacterium tuberculosis* infection in mice. Vaccine.

[4] Williams A, Hatch GJ, Clark SO (2005). Evaluation of vaccines in the EU TB vaccine cluster using a guinea pig aerosol infection model of tuberculosis. Tuberculosis.

[5] Vordermeier HM, Villarreal-Ramos B, Cockle PJ (2009). Viral booster vaccines improve *Mycobacterium bovis* BCG-induced protection against bovine tuberculosis. Infect Immun.

[6] Verreck FAW, Vervenne RAW, Kondova I (2009). MVA.85A boosting of BCG and an attenuated, phoP deficient *M. tuberculosis* vaccine both show protective efficacy against tuberculosis in rhesus macaques. PloS one.

[7] Worku S, Hoft DF (2000). In vitro measurement of protective mycobacterial immunity antigen-specific expansion of T cells capable of inhibiting intracellular growth of bacille Calmette-Guerin. Clin Infect Dis.

[8] Li Q, Whalen CC, Albert JM (2002). Differences in rate and variability of intracellular growth of a panel of *Mycobacterium tuberculosis* clinical isolates within a human monocyte model. Infect Immun.

[9] Marsay L, Matsumiya M, Tanner R (2013). Mycobacterial growth inhibition in murine splenocytes as a surrogate for protection against *Mycobacterium tuberculosi*s (M. tb). Tuberculosis.

[10] Sauerwein RW, Roestenberg M, Moorthy VS (2011). Experimental human challenge infections can accelerate clinical malaria vaccine development. Nat Rev Immunol.

[11] Carrat F, Vergu E, Ferguson NM (2008). Time lines of infection and disease in human influenza: a review of volunteer challenge studies. Am J Epidemiol.

[12] Statler J, Mammen M, Lyons A, Sun W (2008). Sonographic findings of healthy volunteers infected with dengue virus. J Clin Ultrasound.

[13] Marwick C (1998). Volunteers in typhoid infection study will aid future vaccine development. JAMA.

[14] Minassian AM, Satti I, Poulton ID, Meyer J, Hill AVS, McShane H (2012). A human challenge model for *Mycobacterium tuberculosis* using *Mycobacterium bovis* bacille Calmette-Guerin. J Infect Dis.

[15] McShane H, Brookes R, Gilbert SC, Hill AVS, Mmun INI (2001). Enhanced immunogenicity of CD4+ T-cell responses and protective efficacy of a DNA-modified vaccinia virus Ankara prime-boost vaccination regimen for murine tuberculosis. Infect Immun.

[16] Minassian AM, Ronan EO, Poyntz H, Hill AVS, McShane H (2011). Preclinical development of an in vivo BCG challenge model for testing candidate TB vaccine efficacy. PloS one.

[17] Talbot EA, Williams DL, Frothingham R, Talbot EA, Williams DL (1997). PCR identification of *Mycobacterium bovis* PCR identification of *Mycobacterium bovis* BCG. J Clin Microbio.

[18] Meyer J, Harris SA, Satti I (2013). Comparing the safety and immunogenicity of a candidate TB vaccine MVA85A administered by intramuscular and intradermal delivery. Vaccine.

[19] Wallis RS, Palaci M, Vinhas S (2001). A whole blood bactericidal assay for tuberculosis. J Infect Dis,.

[20] Cunha AJLA, Anna CCS, Mannarino R, Labanca TC, Ferreira S, March MFBP (2002). Adverse effects of BCG revaccination: a report on 13 cases from Rio de Janeiro, Brazil. Int J Tuberc Lung Dis.

[21] Dourado I, Rios MH, Pereira SMM (2003). Rates of adverse reactions to first and second doses of BCG vaccination: results of a large community trial in Brazilian school children. Int J Tuberc Lung Dis.

[22] Pathan AA, Minassian AM, Sander CR (2012). Effect of vaccine dose on the safety and immunogenicity of a candidate TB vaccine, MVA85A, in BCG vaccinated UK adults. Vaccine.

[23] SSI Description of BCG VACCINE SSI.

[24] McShane H, Pathan AA, Sander CR (2004). Recombinant modified vaccinia virus Ankara expressing antigen 85A boosts BCG-primed and naturally acquired antimycobacterial immunity in humans. Nat Med.

[25] Tameris MD, Hatherill M, Landry BS (2013). Safety and efficacy of MVA85A, a new tuberculosis vaccine, in infants previously vaccinated with BCG: a randomised, placebo-controlled phase 2b trial. Lancet.

[26] Fletcher HA, Tanner R, Wallis R (2013). Inhibition of mycobacterial growth in vitro following primary but not secondary vaccination with BCG. Clin Vaccine Immunol.

[27] Worku S, Hoft DF (2003). Differential effects of control and antigen-specific T cells on intracellular mycobacterial growth. Infect Immun.

[28] Kampmann B, Tena GN, Mzazi S, Eley B, Young DB, Levin M (2004). Novel human in vitro system for evaluating antimycobacterial vaccines. Infect Immun.

[29] Cheon S, Kampmann B, Hise AG (2004). Bactericidal activity in whole blood as a potential surrogate marker of immunity after vaccination against tuberculosis. Clin Diagn Lab Immunol.

[30] Williams A, Goonetilleke NP, Mcshane H (2005). Boosting with poxviruses enhances *Mycobacterium bovis* BCG efficacy against tuberculosis in guinea pigs. Infect Immun.

[31] Goonetilleke NP, Mcshane H, Hannan CM, Anderson RJ, Brookes RH, Hill AVS (2003). Enhanced immunogenicity and protective efficacy against *Mycobacterium tuberculosis* of bacille Calmette-Guérin vaccine using mucosal administration and boosting with a recombinant modified vaccinia virus Ankara. J Immunol.

[32] (1963). B.C.G. and vole bacillus vaccines in the prevention of tuberculosis in adolescence and early adult life. Br Med J.

